# Healthy Food Access in Low-Income High-Minority Communities: A Longitudinal Assessment—2009–2017

**DOI:** 10.3390/ijerph16132354

**Published:** 2019-07-03

**Authors:** Punam Ohri-Vachaspati, Robin S. DeWeese, Francesco Acciai, Derek DeLia, David Tulloch, Daoqin Tong, Cori Lorts, Michael J. Yedidia

**Affiliations:** 1 College of Health Solutions, Arizona State University, Phoenix, AZ 85004, USA; 2 MedStar Health Research Institute, Hyattsville, MD 20782, USA; 3 Department of Plastic and Reconstructive Surgery, Georgetown University School of Medicine, Washington, DC 20007, USA; 4 Center for Remote Sensing and Spatial Analysis, Department of Landscape Architecture, Rutgers University, New Brunswick, NJ 08901, USA; 5 School of Geographical Sciences and Urban Planning, Arizona State University, Tempe, AZ 85287, USA; 6 Department of Nutrition, Northern Arizona State University, Flagstaff, AZ 86001, USA; 7 Center for State Health Policy, Institute for Health, Health Care Policy, & Aging Research, Rutgers University, New Brunswick, NJ 08901, USA

**Keywords:** food environment, food access, low-income communities, supermarket, grocery store, convenience store

## Abstract

Disparities in healthy food access are well documented in cross-sectional studies in communities across the United States. However, longitudinal studies examining changes in food environments within various neighborhood contexts are scarce. In a sample of 142 census tracts in four low-income, high-minority cities in New Jersey, United States, we examined the availability of different types of food stores by census tract characteristics over time (2009–2017). Outlets were classified as supermarkets, small grocery stores, convenience stores, and pharmacies using multiple sources of data and a rigorous protocol. Census tracts were categorized by median household income and race/ethnicity of the population each year. Significant declines were observed in convenience store prevalence in lower- and medium-income and majority black tracts (*p* for trend: 0.004, 0.031, and 0.006 respectively), while a slight increase was observed in the prevalence of supermarkets in medium-income tracts (*p* for trend: 0.059). The decline in prevalence of convenience stores in lower-income and minority neighborhoods is likely attributable to declining incomes in these already poor communities. Compared to non-Hispanic neighborhoods, Hispanic communities had a higher prevalence of small groceries and convenience stores. This higher prevalence of smaller stores, coupled with shopping practices of Hispanic consumers, suggests that efforts to upgrade smaller stores in Hispanic communities may be more sustainable.

## 1. Introduction

Healthy food access is critical to improve population health [1] and to reduce social inequalities [2]. In longitudinal analyses, positive health outcomes, including lower risk of heart disease and diabetes, have been attributed to community level access to healthy food [3,4]. Conversely, greater exposure to unhealthy food outlets negatively impacts body mass index (BMI) [5]. In the United States, recommendations from the Centers for Disease Control and Prevention (CDC) and the National Academy of Medicine advocate for improving access to and consumption of healthy, safe, and affordable food, and at the same time reducing access to and consumption of calorie-dense, nutrient-poor foods. One strategy for accomplishing these goals is to increase community access to healthy foods through supermarkets, grocery stores, and healthy convenience/corner stores [6,7]. A richer understanding of the availability of healthy foods among population subgroups is critical to targeting and tailoring such interventions.

Disparities in access to healthy food in communities across the United States have been well documented in cross-sectional analyses [8,9,10,11]. Low-income and minority communities, compared to middle/high-income and predominantly white communities, tend to have more convenience stores, which sell predominately highly processed, energy-dense foods with little fresh produce [12,13]. In addition, these same communities tend to have fewer supermarkets, which carry a greater variety of nutritious food, and often at a lower price [14]. Supermarket prevalence may also differ by race/ethnicity, with predominantly white neighborhoods having the greatest access, followed by black communities, and predominantly Hispanic neighborhoods having the fewest supermarkets [10]. These disparities in healthy food access contribute to an urban environment in which healthy food is inaccessible and unaffordable for many of its residents [15]. 

Among the few existing longitudinal studies, Rummo et al. [16], building on the Coronary Artery Risk Development in Young Adults (CARDIA) study and using retrospective data from a commercial data source, found that density of convenience stores decreased over time in higher income neighborhoods and that fast food restaurant density decreased in low-income areas when the white population in those areas increased. Richardson et al. [17], using the same data source, found that neighborhoods that declined in socioeconomic status over the previous 20 years had the highest numbers of convenience stores compared to upwardly mobile, stable high, and stable low socioeconomic status (SES) neighborhoods. Lastly, James et al. [18] examined historical trends in the food environment in their Framingham study cohort, also using retrospective data from multiple sources, and found that over a 40-year period, poorer neighborhoods, compared to higher-income neighborhoods, consistently had higher numbers of fast food outlets. Taken together, these studies suggest that disparities in access to healthy food have persisted over time.

Our study focuses on a comprehensive set of healthy and unhealthy food outlets among low-income populations in Camden, Newark, New Brunswick, and Trenton, New Jersey, United States, and analyzes prevalence trends over a nine-year period by community-level income and race/ethnicity. This period of observation, 2009–2017, was particularly salient. Not only did it span the occurrence of a major recession, but also during this time, substantial public and private investment in initiatives to improve food access were undertaken in the four study cities. Further, our study methodology improved upon and addressed shortcomings of several previous studies. It avoided misclassification bias associated with sole reliance on commercial data sources assembled retrospectively and not verifiable by ground-truthing. Rather, it relied upon multiple sources of data to maximize capture of relevant change, and subjected the data to an established protocol for cleaning, classifying, and confirming the status of outlets in real time [19].

## 2. Materials and Methods 

Data on retail food outlets in the four study cities were matched to census tracts based on the geocoded location of the outlets. Data were collected at six time points, over a nine-year period between 2009 and 2017. Retail food outlet location data were matched with demographic census data.

### 2.1. Sources of Data and Variable Construction

#### 2.1.1. Census Data

Census tract variables were obtained from the American Community Survey [20], which contains five-year estimates of all variables beginning in 2009. For each census tract in the sample, we drew the five-year estimates for total population, proportion of residents by race/ethnicity, and median household income.

Census tract divisions are updated before each decennial census in order to maintain an optimum population size. While the intention is to keep them the same over time so that statistical comparisons can be made from one census to the next, in some cases it is necessary to split, merge, or eliminate census tracts [21]. In the current analysis, 142 census tracts from the four study cities were included in the longitudinal sample from 2009 to 2017. Census tracts in 2009 were adapted to match the census tracts revised in the 2010 census. The 2010 census geographies were used as the benchmark to calibrate the 2009 demographic and socioeconomic variables, such as population, median household income, and ethnicity. Specifically, layers of 2000 census tract data were overlaid with 2010 census tract data to identify the discrepancies between the geographic boundaries given by the two decennial censuses (e.g., additional area added to a 2010 census tract). We adopted distributions at a finer geography, the block group level, to help achieve better interpolation results [22]. For the population variable, the population count for the discrepancy areas was computed by assuming that population within a block group is uniform. For other socioeconomic variables, the interpolation process involved the inclusion of another variable such as population as these socioeconomic variables are derived variables. For example, the median household income estimation for new census tracts used the block group level income information and the associated population to infer the median household income for the new geography. 

#### 2.1.2. Census Tract Income and Race/Ethnicity Classification

For each year of data included in the analysis, census tracts were classified as lower income, medium income, and higher income based on the tertiles of the median household income distribution across all census tracts in the sample. It is noteworthy that each year the average median income of tracts in the highest tertile in our sample (see Table 1) was still lower than the average median household income of all census tracts in New Jersey ($78,376 in 2009 to $80,088 in 2017) [23]. Majority race/ethnicity of each census tract for a specific year was assigned based on the race/ethnicity that composed ≥50% of the tract for that year. Census tracts with no race/ethnicity composing at least 50% of the tract were classified as majority mixed. Given the small number of majority white and majority mixed tracts, these two categories were combined in the analysis. Therefore, for the final analysis census tracts were classified as majority black, majority Hispanic, or majority white/mixed.

#### 2.1.3. Retail Food Outlet Data

Lists of retail food outlets in each of the four cities were obtained from two United States-based commercial data companies—InfoUSA and Trade Dimensions/Nielsen. Stores were categorized as supermarkets, small grocery stores, convenience stores, or pharmacies based on a combination of North American Industry Classification System (NAICS) codes, sales volume, name recognition, and quantities of specific foods stocked by the store. The methodology was developed by Ohri-Vachaspati et al. [19], building on previous literature [24,25]. Stores with less than $1 million in annual sales were classified as convenience stores, as were those from larger chains such as Wawa, 7-11, and QuickChek. Stores with sales volumes between $1 and $2 million that sold at least three of the following four items—five types of fruits, five types of vegetables, fresh or frozen meat, and skim or 1% milk—were classified as small grocery stores, while those that sold less than three of the items were classified as convenience stores. Information on what items were stocked was obtained by calling stores. Supermarkets had sales over $2 million and four or more checkouts. Local and national pharmacy chains and stores identifying as pharmacies were categorized as such. As a final step, data from multiple sources were de-duplicated and checked for misclassifications. Data were available for 2009, 2012, 2013, 2014, 2015, and 2017.

### 2.2. Analysis

**Figure 1:**
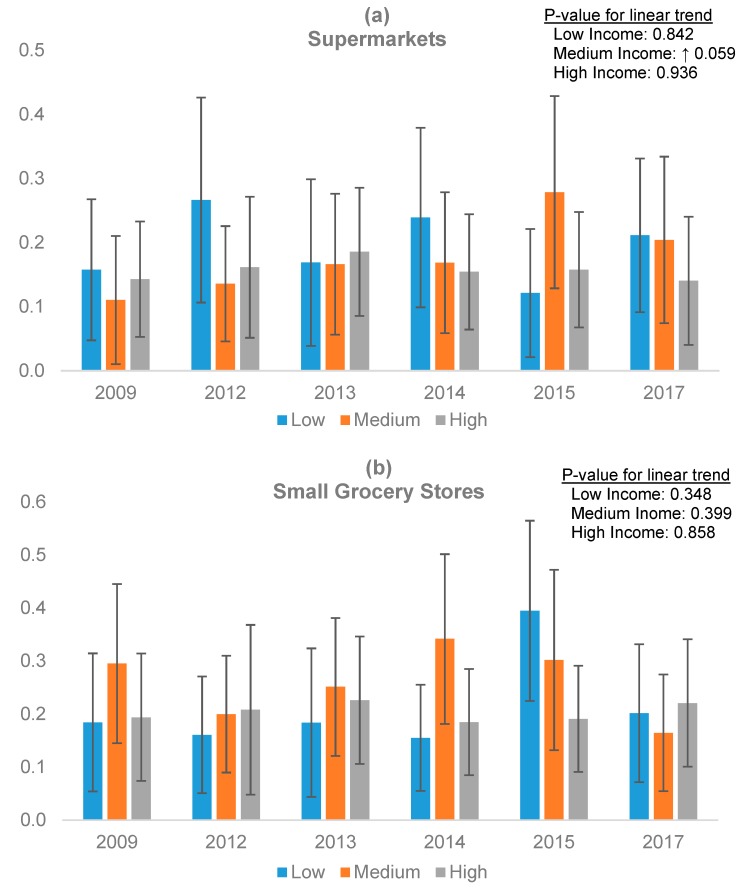

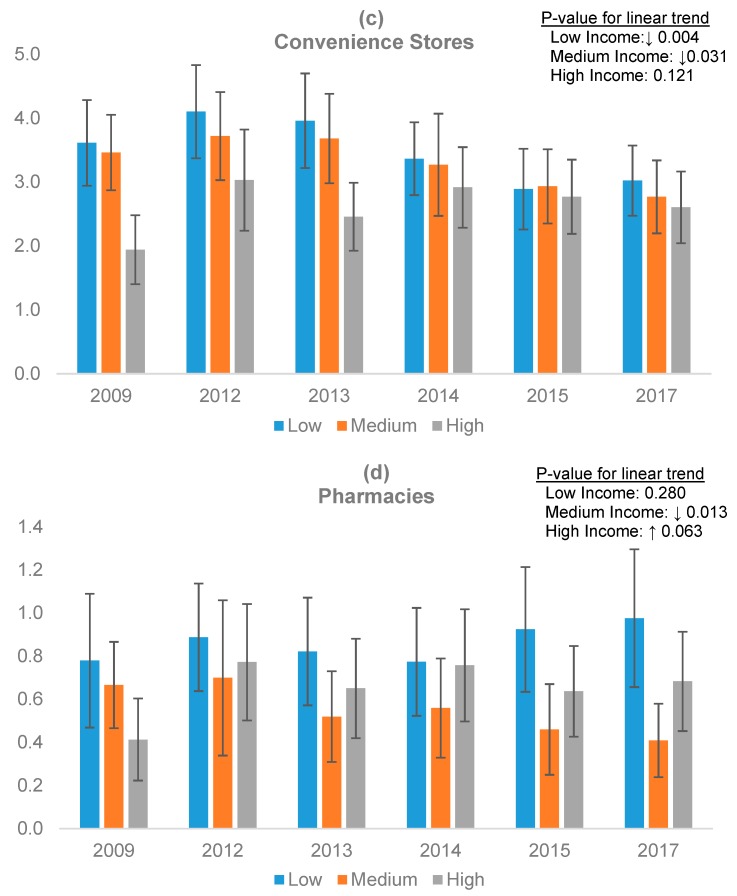
Expected number of (**a**) supermarkets; (**b**) small grocery stores; (**c**) convenience stores; (**d**) pharmacies per census tract by income group. Year-by-year differences: Supermarkets: No differences were observed. Small grocery stores: In 2014 medium-income tracts had a significantly higher number of small grocery stores than did lower- and higher-income tracts. In 2015 small grocery stores were more prevalent in lower income tracts than they were in higher income tracts. Convenience stores: In 2009 and 2013 low-income and medium-income tracts had significantly more convenience stores than did higher-income tracts. Pharmacies: Lower-income tracts had significantly more pharmacies than medium-income tracts in 2015 and 2017. ↓: indicates a declining linear trend over time; ↑: indicates an increasing linear trend over time.

**Figure 2:**
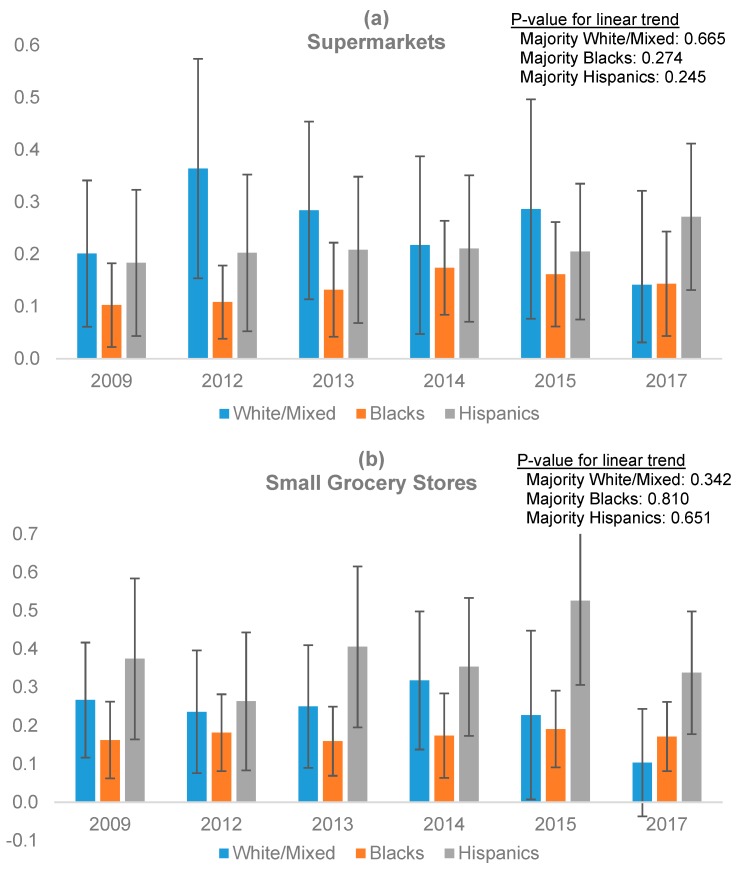

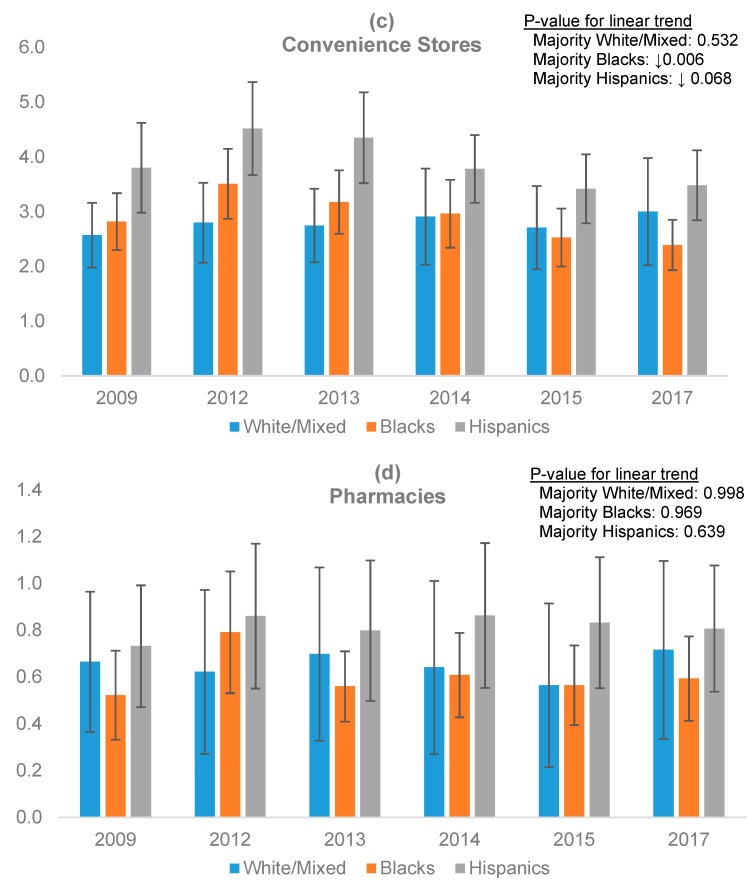
Expected number of (**a**) supermarkets; (**b**) small grocery stores; (**c**) convenience stores; (**d**) pharmacies per census tract by racial/ethnic group. Year-by-year differences: Supermarkets: In 2012 majority white/mixed census tracts had significantly more supermarkets than did majority black tracts. Small grocery stores: Majority Hispanic census tracts had significantly more small grocery stores than did majority black tracts in 2009, 2013, and 2015. Convenience stores: Majority Hispanic tracts had significantly more convenience stores than did majority black tracts in 2009, 2013, 2015, and 2017, and more than majority white/mixed tracts in 2009, 2012, and 2013. Pharmacies: No differences were observed.

All data manipulation and variable construction was done using SPSS-V23 (IBM, Armonk, NY, USA), and statistical analyses were conducted using STATA-V14.0 (StataCorp, College Station, TX, USA). Data for each type of outlet were aggregated to compute counts per census tract. First, distributions of retail store counts and census demographics were examined. Second, bivariate analyses were run to examine the prevalence of different types of stores across census tracts based on their income and race/ethnicity. Third, given that our dependent variables were overdispersed count variables and the measurements were nested within census tracts, negative binomial regression models with clustering at the census tract level were built to examine the trend of the prevalence of different types of stores over time. For each of the four outcome variables (supermarkets, small grocery stores, convenience stores, and pharmacies) we ran two sets of models, one with time as a continuous variable to analyze the linear trend over the entire study period, and one with time as a categorical variable to obtain the year-by-year expected number of stores. In either specification, we first interacted time with tract-level income to examine whether and how the changes in the number of stores affected tracts differently based on their income level. The models adjusted for majority race/ethnicity and total population at the tract level. Then we replicated this set of models but we interacted time with tract-level majority race/ethnicity to examine whether and how the changes in the number of stores affected tracts differently based on their racial/ethnic composition, while adjusting for income and total population. The addition of educational level did not modify the results; therefore, for parsimony it was removed from the models. Results are expressed as mean adjusted store counts per census tract, overall (Table 2), by income (Figure 1), and by race/ethnicity (Figure 2). These adjusted means were obtained from the negative binomial regression models using the ‘margins’ command in Stata.

## 3. Results

Table 1 describes the demographic characteristics of the 142 census tracts included in the analysis. Over the nine-year period, the average median household income (inflation adjusted for 2017) ranged between $25,170 and $21,974 for lower-income tracts, between $40,622 and $32,568 for middle-income tracts, and between $58,392 and $46,544 for higher income tracts. The median household income in majority black and Hispanic tracts declined significantly over the study period (*p* < 0.001 for both), while it increased in majority white tracts (*p* < 0.05) (data not shown). Between 2009 and 2017, most census tracts were majority black (51%–54%), followed by majority Hispanic (26%–34%), and majority white (2%–8%); 11%–15% of the tracts did not have any majority race/ethnic group. Table 2 shows the adjusted mean number of food outlets per census tract, for each year of data, as well as the p-value for the overall linear trend. Over the nine years, the range for supermarkets per tract was 0.14–0.19, small grocery stores 0.19–0.28, convenience stores 2.74–3.58, and pharmacies 0.61–0.77. During the nine-year study period, across the study cities, the number of convenience stores decreased significantly (*p* = 0.008), while the number of supermarkets, small grocery stores, and pharmacies essentially remained the same. 

Figure 1 examines disparities in expected number of stores by census tract income categories for supermarkets (Figure 1a), small grocery stores (Figure 1b), convenience stores (Figure 1c), and pharmacies (Figure 1d) for each year, adjusted for majority race/ethnicity and total population of the census tract. For each type of outlet, statistical significance from the trend analysis, examining linear changes over time, is also shown. Only medium-income tracts showed a slight upward trend in the average number of supermarkets over time (*p* for trend = 0.059); these tracts started with the lowest number of supermarkets at the start of the study period. In year-by-year comparisons, we observed no differences in the number of supermarkets across any tract-level income groups. A significant decline in convenience stores (Figure 1c) was observed over the nine-year period in low- (*p* for trend = 0.004) and medium-income (*p* for trend = 0.031) census tracts, and no change was observed in the higher-income tracts. Disparities were observed in the earlier years (2009 and 2013), where higher-income tracts had fewer convenience stores than did lower-income and medium-income tracts. By the later years of data collection differences in the number of convenience stores among tract-level income groups had disappeared. The average number of small grocery stores per tract did not follow a linear pattern for any income category, despite showing some year-to-year variability. For instance, in 2014 medium-income tracts had a significantly higher number of small grocery stores than did lower- and higher-income tracts; whereas in 2015 small grocery stores were more prevalent in lower income tracts than they were in higher income tracts. We observed a declining trend (*p* = 0.013) in pharmacies in medium income tracts. In 2015 and 2017, there were significantly fewer pharmacies in medium income tracts than in lower income tracts. 

Changes in the food environment over time by race/ethnicity are shown in Figure 2. As with income, expected counts over the nine-year period are presented for supermarkets (Figure 2a), small grocery stores (Figure 2b), convenience stores (Figure 2c), and pharmacies (Figure 2d) by census tract majority race/ethnicity categories. Expected counts of food outlets in these tracts are adjusted by tract-level income and total population. The average number of supermarkets did not change significantly over time in majority Hispanic, black, or white/mixed census tracts. In terms of year-by-year disparities, only in 2012 was the number of supermarkets in white/mixed tracts significantly higher than in majority black neighborhoods. While in census tracts with a black majority there was a significant decline in the average number of convenience stores over time (*p* for trend = 0.006), a marginal decline was observed in Hispanic tracts (*p* for trend = 0.068), with no changes observed in white/mixed tracts. Tracts with a Hispanic majority had significantly more convenience stores than did majority black (2009, 2013, 2015, 2017) and majority white/mixed tracts (2009, 2012, 2013). No significant changes in trends were observed over time in the number of small grocery stores and pharmacies within each racial/ethnic category of census tracts. However, for some of the years, Hispanic tracts had more small grocery stores compared to black tracts (2009, 2013, 2015).

## 4. Discussion

This study examined changes in the food environment over a nine-year period (2009–2017) in four low-income, high minority New Jersey cities in the United States. We found that there were fewer food outlets in 2009, the year coinciding with the great recession in the country. In the subsequent year, the number of stores in most categories increased. After this initial upward trend, the average number of convenience stores per tract declined significantly over time. The decline in convenience store prevalence was more pronounced in lower- and medium-income and in majority black tracts. The marginal increase in supermarkets was restricted to medium-income tracts. Hispanic majority tracts tended to have a larger number of smaller stores—both convenience stores and small grocery stores—over the entire study period. 

Compared to previous longitudinal research [16,17,18], the communities included in the study sample were high minority and low-income. More than half of the tracts across all years had a majority black population and between a quarter and a third had a majority Hispanic population; the proportion of tracts with a white majority and with no majority race declined over time. Even in the highest income tertile in 2017, the average median household income was less than $50,000 for all cities, which is substantially lower than the median household income in the US ($60,366) and in New Jersey ($80,088) [23]. Furthermore, the average household income for the three income tertiles declined over the nine-year period, and these declines were most prevalent in black and Hispanic majority census tracts. Focusing on these types of communities, which are most impacted by limited access to healthy food, is an important addition to the literature. Isolating these neighborhoods for analysis provides more granular findings that can be used to identify effective methods to increase food access, and to pinpoint where, specifically, those efforts should focus.

Previous studies have reported an inverse relationship between neighborhood income and number of convenience stores. In the present study, in the earlier years the higher-income tracts had significantly fewer convenience stores than did lower- and medium-income tracts. However, over time the number of convenience stores declined significantly in lower- and medium-income tracts, while the numbers remained the same in higher-income tracts, resulting in a narrowing of the divide that existed between categories. The reason for the decline may be associated with several factors. In the medium income tracts, a slight, marginally significant increase in number of supermarkets over time was observed, possibly creating greater competition among convenience stores and pulling customers away from these small businesses. The decline may also have been associated with the decreases in income and the market’s response to lower buying-power of neighborhood residents. In the lower income tracts the average median income in 2009 was $25,170 (inflation adjusted to 2017) and dropped to $21,974 in 2017. Similar declines were observed in the other two income categories. Evidence from New York City suggests that the turnover rate for retail establishments is higher in census tracts with a median household income at or below the city median compared to tracts with incomes above the city median [26]. Previous research [27,28] reported that stores in high-poverty census tracts have a shorter survival length. Therefore, in the present study many smaller stores that rely particularly on neighborhood shoppers might have been forced to close without necessarily being replaced, because of the decreasing buying power of nearby residents.

Secondarily, in all four communities efforts were underway to upgrade small stores and provide resources to help them carry healthier options [29,30]. Successful upgrades may have resulted in convenience stores being reclassified as small grocery stores if they met the criteria of carrying the desired types and amounts of healthy options; however, given the limited number of small grocery stores in each tract, these conversions would have made only a small contribution to changes in the number of convenience stores over time. 

Lower-income neighborhoods had a higher prevalence of pharmacies over the years, and the numbers were significantly higher when compared to medium-income tracts in the later years of analysis. Although pharmacies are not often considered as part of the food access conversation, snack foods have become ubiquitous in these stores [31]. Foods such as candy, sweetened beverages, salty snacks, and baked sweets are present within arm’s reach of the cash register in 96% of pharmacies in all regions of the US [32]. Many chain pharmacies dedicate substantial aisle space to snack foods [33]. While pharmacies play an important role in communities, the combination of increased access to both convenience stores and pharmacies observed in the lowest income census tracts in the current sample may further expose low-income residents to abundant energy-dense, nutrient-poor foods. Further, current efforts to improve healthy food access in small stores often do not target pharmacies.

This study covered a period during which a number of initiatives aimed at improving access to healthy food outlets were under way nationally and locally in the study communities, including opening new supermarkets [34,35]. It is possible that these efforts contributed to the small increase in the number of supermarkets in middle-income tracts, which started with the lowest number of supermarkets in 2009. 

With regard to race, Hispanic tracts had a higher number of convenience stores and small grocery stores compared to majority black and mixed/white tracts. A significant decline in number of convenience stores was observed in majority black tracts and to a lesser extent in majority Hispanic tracts. In spite of this decline, Hispanic majority tracts still had the highest prevalence of convenience stores for all years. Larger numbers of small stores in Hispanic communities may be a function of the market’s response to differences in shopping patterns between Hispanics and non-Hispanics. Compared to non-Hispanics, Hispanics tend to purchase more grocery items at convenience stores [36,37] and, compared to blacks, also shop at stores closer to home. Results of a 2009 survey in three of the four study cities demonstrated that Hispanic households were more likely than were non-Hispanic black households to do most of their shopping inside their neighborhood. In particular, about half of non-Hispanic black households reported shopping within the neighborhood most of the time, as compared to between 60% and 79% of Hispanic residents [38,39,40]. Neighborhood stores in Hispanic communities have also been shown to meet the specific needs of their residents; for example by carrying culturally preferred fruits and vegetables [41].

The higher prevalence of smaller stores, both convenience and small grocery stores, in Hispanic neighborhoods observed in this study, combined with research on shopping patterns and preferences of different race/ethnicity groups [37,41,42] provides insights for design and implementation of interventions aimed at improving the healthfulness of inventory offered by small stores. Specifically, establishing upgrades in small stores in these neighborhoods may hold greater potential for increasing the consumption of healthy foods among neighborhood residents, especially when located in high-traffic areas [43]. Currently, literature is mixed on the success of corner store upgrade interventions [44,45,46]. Future studies may selectively target Hispanic communities where small stores are likely to thrive, and where shopping patterns of community members are more likely to support upgraded stores.

Our analysis shares with prior research a focus on location of food outlets, and does not incorporate data on shopping behaviors; residents may travel across census tracts to purchase food. Another limitation is that, while we used a systematic protocol [19] to ensure that stores were properly categorized, misclassification of some stores can still occur. However, because the classification process remained the same across all years, misclassifications would not differ systematically across years or across income and race/ethnicity categories; therefore, our comparisons remain valid. Other methods of longitudinally examining changes to the food environment (e.g., following each tract over time, regardless of changes in income and racial/ethnic composition) are possible and we have considered them. However, we opted to analyze prevalence trends over time by census tract income and race/ethnicity categories to examine change in disparities over time. The results from these analyses are specific to low-income high-minority census tracts and may not be generalizable to higher income and less-diverse communities.

## 5. Conclusions

The nine-year period between 2009 and 2017 saw significant changes in the food environment in low-income high-minority communities in New Jersey. Significant declines in the prevalence of convenience stores were observed in the lowest-income and highest-minority neighborhoods, most likely resulting from declining incomes in these neighborhoods. A small but significant increase in the prevalence of supermarkets in middle-income tracts may have been a result of efforts to promote healthy food access in the communities under investigation. Our results suggest that communities similar to those we studied that support initiatives to promote healthier inventory in small stores may wish to target Hispanic neighborhoods as they have a higher prevalence of convenience and small grocery stores, and residents of these communities are more likely to shop at these establishments.

## Figures and Tables

**Table 1:** Demographic characteristics of the 142 census tracts included in the analysis of food access in four New Jersey cities.

Census Tract Characteristics	2009		2012		2013		2014		2015		2017
**Census tract median household income ^a^ ($)**	**Actual**	**IA ^b^**	**Actual**	**IA ^b^**	**Actual**	**IA ^b^**	**Actual**	**IA ^b^**	**Actual**	**IA ^b^**	**Actual**
Lower income tracts (lowest tertile)	21,977	25,170	22,413	23,980	22,794	24,026	21,842	22,639	21,630	22,380	21,974
Medium income tracts (middle tertile)	35,468	40,622	34,237	36,632	34,116	35,959	33,951	35,189	32,154	33,269	32,568
Higher income tracts (highest tertile)	50,984	58,392	47,224	50,527	46,902	49,436	45,791	47,461	45,155	46,721	46,544
**Census tract majority race/ethnicity *n* (%)**											
Majority non-Hispanic black tracts	72 (51)		73 (51)		76 (54)		75 (53)		74 (52)		76 (54)
Majority Hispanic tracts	37 (26)		42 (30)		41 (29)		44 (31)		48 (34)		48 (34)
Majority non-Hispanic white tracts	11 (8)		7 (5)		5 (4)		4 (3)		3 (2)		3 (2)
Majority mixed tracts	22 (15)		20 (14)		20 (14)		19 (13)		17 (12)		15 (11)

^a^ For median household income we are reporting the average income within each tertile. ^b^ IA: Inflation adjusted—adjustment factor for 2017 purchasing power.

**Table 2:** Number of stores per census tract—mean (standard error) ^a^.

Store Type	2009	2012	2013	2014	2015	2017	*p* for Trend
Supermarkets	0.14 (0.03)	0.18 (0.04)	0.18 (0.03)	0.18 (0.03)	0.19 (0.04)	0.18 (0.04)	0.213
Small grocery stores	0.23 (0.04)	0.19 (0.04)	0.22 (0.04)	0.23 (0.04)	0.28 (0.04)	0.19 (0.04)	0.942
Convenience Stores	3.03 (0.19)	3.58 (0.21)	3.34 (0.20)	3.12 (0.20)	2.77 (0.17)	2.74 (0.17)	0.008
Pharmacies	0.61 (0.07)	0.77 (0.09)	0.65 (0.07)	0.68 (0.07)	0.64 (0.07)	0.67 (0.07)	0.809

^a^ Models adjusted for racial composition, median household income, and total population at the census tract level.
